# Editorial of Special Issue “The Role of Vitamin D in Human Health and Diseases 3.0”

**DOI:** 10.3390/ijms25137170

**Published:** 2024-06-29

**Authors:** Francesca Silvagno, Loredana Bergandi

**Affiliations:** Department of Oncology, University of Torino, Via Santena 5 bis, 10126 Torino, Italy

After the successful collection of studies published in the past two Special Issues on the role of vitamin D in health and disease, this Special Issue, titled “The Role of Vitamin D in Human Health and Diseases 3.0”, presents another assortment of excellent contributions investigating the mechanisms of action and the impact of vitamin D in a variety of physiological and pathological conditions. Four research articles and a compilation of six reviews were published on the topic of the different aspects of vitamin D activity, including the association between its defective signaling or low levels and many diseases. The research articles, in particular, focused on the molecular mechanisms mediating the anti-inflammatory and metabolic activity of vitamin D, as detailed below.

Carlberg proposed an interesting study of gene modulation in peripheral blood mononuclear cells [1]. This model was useful for dissecting the molecular response to a vitamin D supplementation in vivo, after a bolus of cholecalciferol was given to healthy subjects and blood cells were extracted to analyze the response to endogenously converted active form of vitamin D. The anti-inflammatory general role of calcitriol was highlighted in this study by the discovery that vitamin D downregulates several players of the signaling pathways of HIF-1, TNF-α, TGF-β, and NF-kB, which have a central role in the inflammatory response caused by oxygen deprivation, infection, dysmetabolism, and oxidative stress, respectively. Additionally, a research article by Yakout et al. demonstrated the anti-inflammatory properties of vitamin D supplementation [2], discovering that the correction of vitamin D deficiency in Saudi adults abated the levels of the free radical nitric oxide (NOx), increased carboxypeptidase N2 levels, and improved lipid profiles by increasing HDL cholesterol. NOx can modulate the expression of inflammatory proteins via its regulation of the transcription factor NF-kB; therefore, the inhibitory activity of vitamin D on NOx production can mediate its anti-inflammatory effects. The study suggested that by limiting oxidative-stress-induced inflammation, vitamin D can improve lipid metabolism, especially in males. Vitamin D deficiency was also studied in a population of obese women. Considering how obesity and inflammation are strictly related to low levels of this hormone, an extremely relevant study from Mirza et al. [3] revealed that vitamin D deficiency may contribute to obesity-related vascular dysfunction by inducing inflammatory adipokine expression through hypomethylation. These interesting results not only deepen our knowledge about the mechanisms through which vitamin D protects against inflammation but also show the evidence of its epigenetic influence. Indeed, the observation that vitamin D levels are strongly associated with DNMT1 and TET1 protein expression and that vitamin D demonstrated a robust positive association with DNA methylation of inflammatory adipokines opens new avenues for future mechanistic studies testing the interaction between vitamin D and DNA methylation in tissues.

Very intriguing and novel details of the metabolic activity of vitamin D in cancer were unveiled by the experimental work of Piotrowska et al. [4]. Their research on melanoma is greatly relevant, since low levels of vitamin D measured at the time of melanoma diagnosis is associated with thicker tumors and poorer prognosis. This study showed that vitamin D potentiated the abatement of mitochondrial respiratory parameters and enhanced the cytotoxicity of chemotherapy in a pre-clinical model of melanoma, supporting the efficacy of vitamin D as adjuvant agent in the treatment of malignant melanoma.

In addition to these, the reviews published in this Special Issue discuss the impact of hypovitaminosis D in health and its associated diseases, highlighting the critical issues of supplementation protocols in particular.

Similarly to the above-mentioned research articles that dissected the molecular basis of the association between vitamin D insufficiency and dysmetabolic conditions, further evidence was described in a broad review [5] that supports the notion that vitamin D levels are associated with types 1 and 2 diabetes mellitus, metabolic syndrome, cardiovascular disease and gestational diabetes mellitus. The latter is not the only complication of pregnancy associated with vitamin D deficit. To further this subject, the consequences of hypovitaminosis D during gestation are timely addressed in two other reviews. The first of these deals with vitamin D metabolism in pregnancy [6], which presents peculiar characteristics necessary for calcium-dependent fetus growth and maternal–fetal immunological tolerance; furthermore, the consequences of hypovitaminosis and supplementation in pregnancy and breastfeeding are discussed. The focus of the second study is centered on the association of vitamin D deficiency with preeclampsia development [7], which is a debated theme due to the contradictory observations presented by current research. In this review, the authors summarize and analyze data on the effects of 25(OH)D3 deficiency and its supplementation on pregnancy, labor and fetal and neonatal outcomes.

The impact on bone metabolism and calcium–phosphate homeostasis is the most documented beneficial effect of the vitamin D hormone on health. However, in several bone-related diseases, the role of vitamin D and the outcome of its supplementation are still controversial or have not yet been properly investigated. In this collection of papers, two studies focused on bone remodeling. A scoping review was conducted to assess the incidence of vitamin D deficit in osteogenesis imperfecta (OI) [8]. Unfortunately, the limited number of studies and the heterogeneity of the collected data made it difficult to reach general conclusions, although the authors observed that low levels of vitamin D are frequently found in OI patients. In the analyzed studies, vitamin D supplementation was carried out by heterogeneous protocols, such as the association with drug therapy and calcium intake, and in any case, did not change the risk of fracture—the primary goal in the treatment of OI. For these reasons, vitamin D can presently be considered part of a multidisciplinary approach in the treatment of OI. On the other hand, a second review [9] summarized the most recent information on the cross-talk between vitamin D and the key regulator of serum phosphates, fibroblast growth factor 23 (FGF23), in the pediatric population under normal conditions, as well as in several diseases such as rickets, chronic kidney disease and hypophosphatemic disorders.

Lastly, a systematic review and metanalysis revealed that serum vitamin D concentrations are influenced by the type of training [10]. Because it was observed that outdoor training is not always associated with higher vitamin D concentration than indoor exercise, the authors of the study in question concluded that supplementation to correct vitamin D deficiency in athletes should not only be based on the type of training alone.

Overall, this Special Issue contains an interesting combination of articles that present novel molecular data on the mechanisms of action of vitamin D and a broader landscape of its anti-inflammatory properties that are lost in several diseases in the event of deficiency. The analyses presented in these works highlight how the efficacy of restoring vitamin D levels is limited by the heterogeneity of the patients [2,5,8,9], administration protocols [6,8,9] and individual response to the hormone [1].
